# Small businesses and their challenges during COVID-19 pandemic in developing countries: in the case of Ethiopia

**DOI:** 10.1186/s13731-021-00191-3

**Published:** 2022-01-10

**Authors:** Abriham Ebabu Engidaw

**Affiliations:** Management Department, Woldia University, Woldia, Ethiopia

**Keywords:** COVID-19 pandemic, Small business, Challenges of small business

## Abstract

Mainly this study was aimed at demonstrating the challenges of small businesses during the corona virus pandemic in developing countries, specifically in Ethiopia. Methodologically, the study deployed secondary data analysis following a descriptive research design. Furthermore, this study was conducted through both qualitative and quantitative research approaches. The secondary data analysis result shows that many small and large businesses are suffering challenges and this unprecedented coronavirus crisis has caused destruction for many businesses in the globe and it is challenging to survive with reduced revenue, jobs lost and life slowing down and weak marketing performance even difficult to keep a calm head and their business alive. To support these businesses, the government should use different mechanisms by cooperating with wealthy peoples and other non-governmental organizations. Besides, small business owners should manage expectations and communicate with staff, suppliers, banks, and customers throughout this coronavirus frightening. Reduce expenses be open to their employees about their finances and keep marketing, use different alternatives to deliver their product, and recover from the crisis.

**Supplementary Information:**

The online version contains supplementary material available at 10.1186/s13731-021-00191-3.

## Introduction

COVID-19 is shuddering the world economy and it is a pandemic making a giant distractions to life and livelihoods as well as social and economic systems in the world. Based on different reports, it is the most horrible global crisis since WW II. This virus is highly transmittable and has spread with inconsistent progress in every corner of the world without any variance. COVID-19 is a massive health crisis BUT also much more. It is a systemic shock with profound implications, both in the short- and medium- to long-term. This virus has triggered a substantial short-term economic contraction, shuttered many firms, whether big or small, thrown tens of millions out of work, and has other effects on business activities. To prevent unemployment, poverty, and food insecurity rates from further skyrocketing during any time, small and medium enterprises around the globe can and should play a crucial role.

Small businesses are the backbone of any economy, and with the ripple effect of COVID-19 on economies all over the world, their protection has become important more than ever. Since the first case of pandemic surfaced in Ethiopia, the government has been taking various sweeping health and economic measures to mitigate its impact. Recognized by the government as a driver for economic growth and job creation, small businesses, or more commonly referred here as small and micro enterprises as the lexicon goes, the sector has been growing steadily for the past decade or so. However, facing the wrath of the coronavirus pandemic, most of these firms face difficulty surviving in the current climate for even above 5 months, Ethiopian press agency (2020).

According to ONE UN Ethiopia (2020) assessment report, Sub-Saharan Africa (SSA), including Ethiopia, are unlikely to escape the direct and indirect effects of the pandemic and the attendant global crisis. While the trajectory of COVID-19 is still at its initial stages in the region, the repercussions of development elsewhere are already being felt strongly. In these circumstances, it is vital to understand the scale, nature, and depth of social and economic impacts to design an appropriate and effective policy and programmatic response, whether at the country, regional, or global levels. Ethiopian small business also faced some major challenges as it was struck by COVID-19. The macroeconomic and development situation was challenging, evident in slowing but still high growth, the risk of debt distress, low levels of domestic resource mobilization, high inflation, high unemployment, not least among youth trying to find opportunities in an economy with elevated levels of informality, low forex reserves and significant pressure on the exchange rate of the Birr. Social unrest, triggered by longstanding issues that could now be aired in a more open civic and political environment, has led to conflict, loss of lives and property and, at the last count, 1.7 million internally displaced persons (IDPs). MSMEs not only play a vital l role in providing employment opportunities but also contribute to the socio-economic development of the country, notably in their role as facilitators for the transition to an industrial society. Amongst the several things this pandemic has taught us, there is the need to modernize the economy, including surveying the small business sector and identifying both the formal and informal sectors at sub-city level in the country and the small businesses themselves have also a part to play. While some businesses are taking steps to safeguard their business or are pausing their plans to grow, others are pivoting to other branches by gauging local demand even as an opportunity, like those that have switched to producing sanitizers, facemasks and other preventive materials.

According to Fairlie (2020a), find that the number of working business owners plummeted from 15.0 million in February 2020 to 11.7 million in April 2020 because of COVID-19 mandates and health- and economic-driven demand shifts. The loss of 3.3 million active business owners (or 22%) was the largest drop on record. When conditioning on working roughly 2 days per week or 4 days a week, the losses are even larger (28% and 31 percent, respectively). Total hours worked by all business owners dropped by 29%. Although incorporated businesses are more growth-oriented and stable, they experienced a drop of 20% from February to April 2020. The findings indicate that there was a partial rebound from April 2020 numbers in May and an additional rebound in June. The number of active business owners bounced back by 7 percentage points resulting in a 15% drop in business activity from February to May 2020, and an additional 5 percentage points rebound in June resulting in an 8% drop in business activity from February to June 2020.

These results build on the findings from a few related studies of the early effects of the coronavirus on small businesses in different countries. Employer business applications as measured by the U.S. Census weekly Business Formation Statistics (BFS) fell in 5 weeks from mid-March to mid-April by over 27% relative to the previous year (Wilmoth, 2020). Examining more recent data from the BFS there is some evidence of a bounce back, but weekly estimates show a lot of variation (U.S. Census Bureau, 2020). Estimates from the weekly U.S. Census Small Business Pulse Survey indicate that roughly 50% of businesses report having a large negative effect from the COVID-19 pandemic and that only 15–20% of businesses have enough cash on hand to cover 3 months of operations (Bohn et al., 2020; U.S. Census Bureau, 2020). Another weekly survey indicates that decreased demand is more problematic than supply factors, such as accessing materials and goods (Desai & Looze, 2020). Bartik et al. (2020) conducted a survey in late March of nearly 6000 small businesses that were members of the Alignable business network. They find that 43% of businesses are temporarily closed, large reductions in employees, and the majority of businesses have less than 1 month of cash on hand.

Alexander et al. (2020) research examined the financial fragility of many small businesses, and how deeply affected they are by the current crisis. In their sample, which is skewed toward the retail sector, they found that 43% of businesses were temporarily closed and that employment had fallen by 40%. This represents a shock to America’s small firms that has little parallel since the Great Depression of the 1930s. The study result suggest that many of these firms had little cash on hand toward the beginning of the pandemic, which means that they will either have to dramatically cut expenses, take on additional debt, or declare bankruptcy. This highlights the ways in which the immediacy of new funding might impact medium term outcomes.

Based on the above problem statements and lack of sufficient findings on the related area the researcher was initiated to conduct this study. So that the study will fill the knowledge and other gaps such as researches with inconsistent results that are conducted in the recent world.

### Crisis and small business firms

There’s no doubt that the COVID-19 pandemic has added to small business challenges around the world, regardless of size, location, or funding. According to Eggers (2020), most of the studies that focus on finance are concerned with the consequences of the crisis on small firms, namely, the lack of funding and financing sources. The strategy-oriented studies indicate that successful firms adopt a strategy that is both market- and entrepreneurship-oriented during a crisis. Small business research has recognized the importance of a crisis perspective (Herbane, 2010). A recent review of literature on crisis and small- and medium-enterprises (SMEs) finds that most of the publications focus on financial issues (51%), followed by strategy (41%), and institutional environment (8%) (Eggers, 2020). Moreover, based on research conducted after the 2004–2012 economic crisis about entrepreneurial culture and the knowledge diversity of small firms in the United Kingdom (Bishop, 2019), Kuckertz et al. (2020) argue that adequate entrepreneurial responsiveness cannot be addressed by short-term measures and needs consistent policies. This highlights the importance of considering the temporal perspective of the crisis. A recent qualitative study about the effect of the COVID-19 pandemic on 16 startups in Germany (Kuckertz et al., 2020) examines how innovative startups deal with the lockdown and the most effective policies. They find that many startups deploy various responses associated with resilience to turn crisis-induced adversity into opportunity. They propose that entrepreneurs who demonstrate flexibility in their business models are likely to access broader emerging opportunities. This finding points to the temporal aspects of the crisis that require further investigation.

### Small business challenges to adapt to the ongoing crisis

One of the biggest trends to emerge during the COVID-19 pandemic is small businesses going online and creates different opportunities to solve many challenges. Indeed, for many small businesses, the internet remains a lifeline, helping them to stay afloat during the pandemic.

According to Facebook’s report, in the 30 days prior to the survey fielding, 23% of businesses reported using digital ordering tools, 16% service delivery tools, and 37% digital payment tools. 36% of operational personal businesses that use online tools report that they are conducting all their sales online. However, we have also seen a lot of small businesses find creative ways to succeed during COVID-19, from expanding into new markets to finding new ways to deliver their products and services. At the same time, a number of large technology companies such as Facebook and Google are creating new ways for small businesses to connect with their customers.

Even when businesses remain open, employees are experiencing financial cuts from lost employment or fewer hours worked. It’s important to remember that any time a business closes or has to lay off workers, it affects entire communities of people who rely on income from jobs to support themselves, and in turn, support other local businesses and organizations.

## Study objectives

The main objective of this study was to investigate the small business challenges during the corona virus pandemic in Ethiopia. In pursuance of this objective, the following research questions were administered:

1. Do small businesses have challenges in operating their business during the pandemic? What are they?
2. To what extent has these challenges affected their operations?

## Study significance

This study contributes to the existing literature in crisis management and identifies the survival and resilience strategies of small businesses during a long-lasting crisis and challenge. I also provide recommendations for small businesses on how to remain flexible or competitive through resilience and renewal strategies, and the researcher has also gave relevant recommendations for policymakers and other concerned bodies.

## Methodologies

This study used a descriptive research design and employed a mixed approach that means the study was conducted through both a qualitative and quantitative research approaches. The target population of the study were small business firms operating in Ethiopia during the corona virus pandemic starting from January 2011- up to September 2020 period. The data analysis technique used under study was the descriptive analysis method and time serie data analysis, because the researchers used secondary data analysis relating to the challenges of these businesses during the COVID-19 crisis.

## Results and discussion

### Secondary data analysis and literature reviews

Based on the WHO’s report, the COVID-19 crisis was established in China in December 2019 and soon after became a global difficult pandemic. As of September 24, 2020, 213 countries and territories around the world were affecting, a total of 32,298,738 people infected, 984,974 deaths, and 23,820,147 patients recovered. Of these, 7,493,617 are active cases or currently infected patients (Worldometers, 2020). Although, crises can be highly damaging for business as they erode trust, damage company value, threaten business goals and objectives, and may even lead to business failure. Existing literatures suggest that small rmss may be more vulnerable to crisis events due to lower levels of preparedness, resource constraints, relatively weak market positions, and higher dependence on government and other domestic agencies. SMEs usually suffer from high losses, reduced sales volume, inability of meeting contract terms, reduction in staff numbers, and even close down of the business during or after crises. During this kind of challenging times, new startup firms have a high chance of surviving during crisis periods than during the growth period, likely due to the lack of job opportunities. Entrepreneurship activities could offset the negative impacts of crises by maintaining the flow of goods and services and restoring the public condense of other business owners and the community at large, and entrepreneurs pursued new opportunities and established new directions for their firms during crises.

Based on these findings, post-crisis organizational learning capability is also critical to recovery. SMEs with strong dynamic and innovative capabilities and are willing to learn from crises events recover quickly (Boin, 2008, Saunders et al., 2014). Similar findings were obtained by Bullough and Renko (2018), who stated that entrepreneurs should engage in business development training and seek networking events or special lectures to learn by modeling others who have survived through challenging times.

Ethiopia can be considered as a high-risk country based on the societal structure and socioeconomic basis. There are strong social ties and attachments with frequent physical interactions that have been developed for centuries as a beneficial means of integrity, which are now considered risk factors for COVID-19 transmission. In urban cities such as Addis Ababa, institutions that provide public services are inadequate, such that crowding is common in hotels, cafes, restaurants, public transportation, market places, hospitals, and other social institutions. These may find it difficult to maintain physical/social distancing as a means of infection prevention strategy, thus may facilitate swift spread of coronavirus disease and run business activities.

### COVID-19 in Ethiopia

**Fig. 1:**
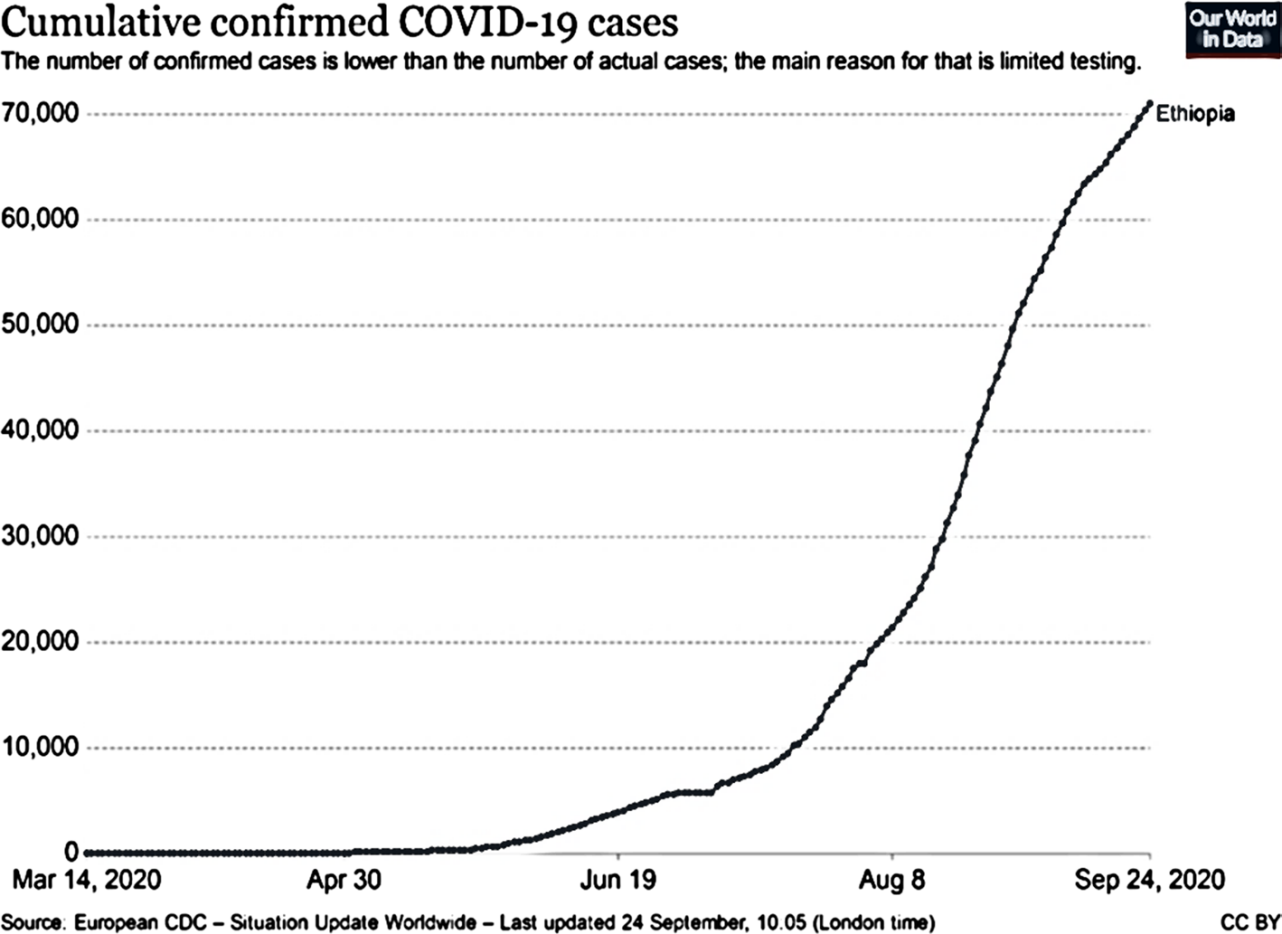
Cumulative confirmed cases of COVID-19 in Ethiopia, up to Sep 24, 2020

**Fig. 2:**
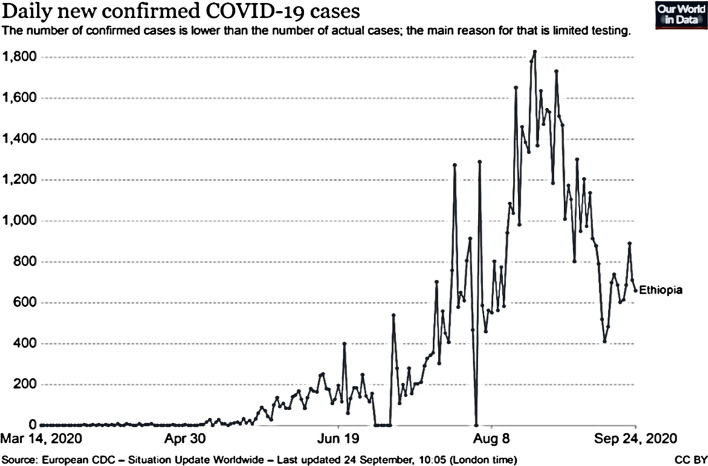
Daily new confirmed COVID-19 cases up to Sep, 24, 2020

Based on the above two figures, COVID-19 is spreading in a fast way in the country starting from April up to present. This pandemic’s spread has created a great challenge on different small businesses performance and peoples day-to-day activities, this will affect the country’s economy as well as the individual life of the society (Figs. 1, 2).

In Ethiopian there is limited testing and it is difficult to know who is infected or not, even with this limited testing the virus is transmitting overindulgently with above 70,000 cases up to September 24, 2020.

As indicated by different researchers, MSMEs are viewed as seedbeds for the growth of big enterprises. The socio-economic impacts being felt across Ethiopia already are wide-ranging and serious, with the potential to become severe, depending on the combination of the pandemic’s trajectory, the effects of countermeasures and fundamental and structural factors. These impacts are summarized below.

### Most impacted groups, sectors and geographic areas by COVID-19 in Ethiopia

In the country, specially, employees working in micro, small and medium-size enterprises (MSMEs) in the urban and rural sectors including (manufacturing, construction, trading, retail, hospitality, and tourism), predominantly helpless children and adolescents such as street children, workers in industrial parks who are already laid off or in danger of losing their jobs, frontline health care professionals, Children of school-going age who are from poor, food insecure households, groups with specific vulnerabilities (persons living with HIV/AIDS, persons with disabilities, older persons, the homeless), migrants, returnees/relocates and returning migrants, Developing regional states (DRS), MSMEs in supply chains in construction, manufacturing, agro-industry, hospitality, tourism, and retail, MSMEs in supply chains for agricultural and horticultural exports as well as production + marketing of critical food crops and soon.

### Key issues and COVID-19 impacts

*(United Nations Economic Commission for Africa) (UNECA *2020*):*

#### MSMEs

30% + of Ethiopia’s SMEs could be in jeopardy, mostly in urban areas and those embedded in small-scale manufacturing, export, construction, and service industry supply chains.

#### Jobs

A 10–15% loss of employment/livelihoods leading to a cumulative loss of perhaps 1.6–2.4 million jobs/livelihoods depending on the severity and duration of the crisis, mostly in urban areas. In the worst case, 3.2–4 million could lose their jobs/livelihoods. Knock-on effects on small businesses/enterprises and self- as well as wage employment in both the formal and informal sectors, especially in the services sector, affect larger urban centers most sharply with business closures and rising unemployment or loss of livelihood and loss of productivity in the case of widespread illness in the workforce.

### Sectorial/sub-sectorial impacts

#### Agriculture

Production might drop by 30% if producers revert to the extensive production system for cash crops. Significant income losses in specific sectors, e.g., livestock, horticulture and supply chain disruptions, are an increasing possibility. Start-up of agro-industrial parks will be delayed and broader negative impacts on food availability, access, and utilization.

#### Construction

Under severe pressure, likely to be one of the most impacted sectors.

#### Manufacturing

Total shutdown or sharp drop in production capacity and reduced employment in industrial parks, subsectors such as textiles and garment and leather and leather products will be hit hard, the flower industry faces catastrophic losses and agro-food processing but beverages subsector will be relatively less impacted.

#### Services (tourism, hospitality, aviation, trading, retail)

High likelihood of closure of businesses and large-scale loss of jobs/livelihoods, in both the formal and informal sectors, especially in urban areas, women, who are disproportionately represented in the informal sector, will be impacted seriously and significant and prolonged forex losses from tourism are very likely.

#### Education

The COVID-19 pandemic has resulted in school closures across the world. It is estimated that learning for 89% of the world’s student population has been disrupted. In Ethiopia, schools have been closed since 16 March 2020 and this is likely to remain the case until the end of the academic calendar. This means that over 26 million children are currently not in school, of which approximately 77% are primary school pupils. These children are neither learning nor benefitting from other school-based support mechanisms, such as protection, health, and school feeding. In short, children’s well-being is at risk. Key education indices which were already dismal before the COVID-19 pandemic are at risk of worsening in the current crisis.

## Estimations of impact of corona virus

*(Based on ONE UN Assessment of Socio-economic Impact of COVID-19 in Ethiopia, *2020*)*

Response and recovery will also have a higher likelihood of success if they:

- Promote measures that put people at the center and protect them and their rights While also conserving vital economic and financial assets and systems;
- Recognize and target those sectors and groups that are most severely impacted and are either already or likely to be left behind;
- Avoid distortions in policy and investments that turn temporary measures into permanent ‘giveaways’ unless deliberately designed as incentives connected to longer term development objectives; and.
- Seize the opportunity to boost longer term goals tied to the short term goals that foster a fairer and more resilient, productive, greener, and sustainable future for Ethiopia.

**Fig. 3:**
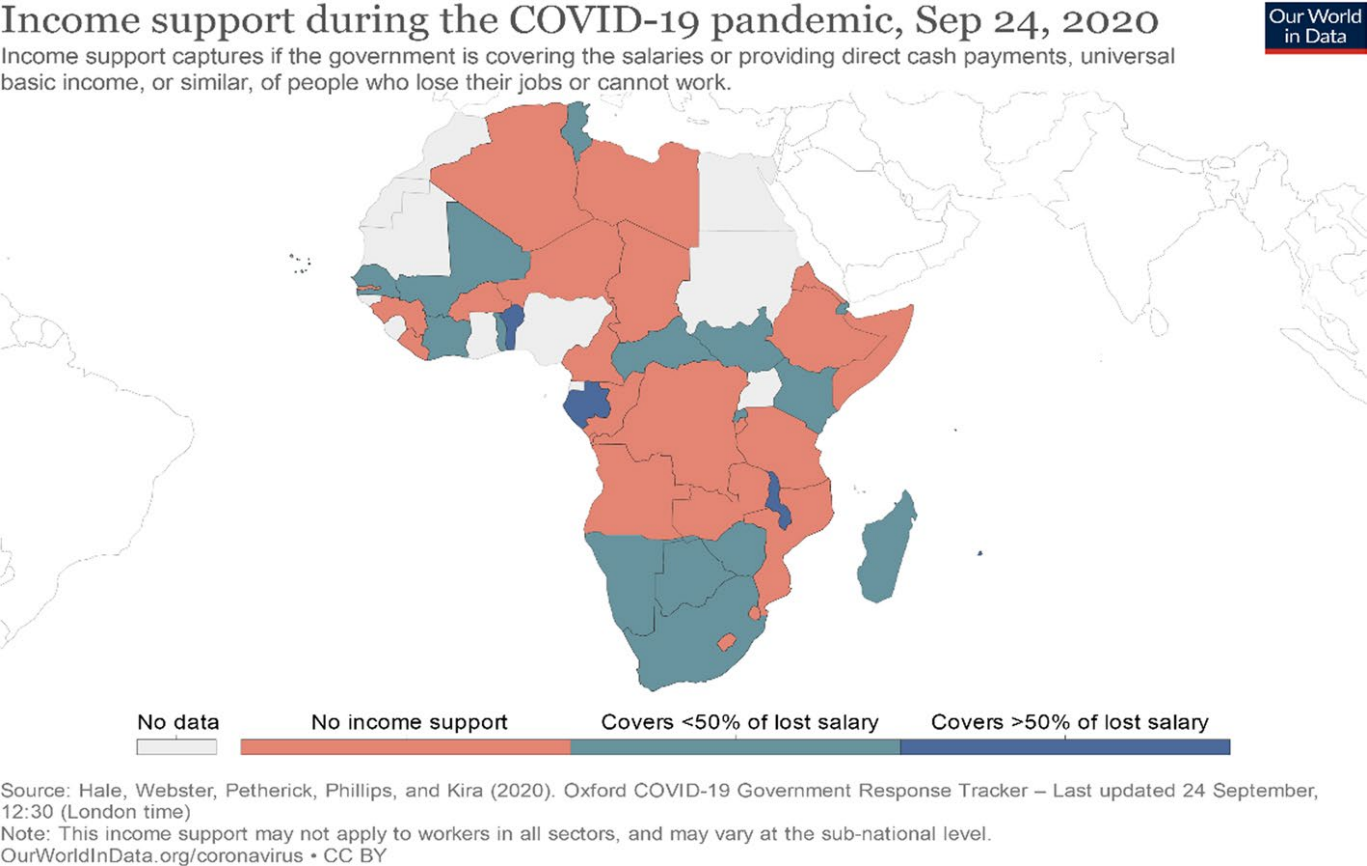
Income support during COVID-19

As clearly shown in the above Fig. 3, in Ethiopia there is no income support for workers and small business firms who lost their job because of different economical and other reasons, but this is not an impossible task, because Ethiopia already has several of the policy instruments, tools, institutions and programs needed to act now and act effectively. There are policy options that can be designed and delivered in Ethiopia as part of its own home-grown answer to the need for immediate response and accelerated recovery. Funding can also be found, partially, if not fully, from some adjustments in budgetary allocations and incentives, regulatory changes, and substantial, although still insufficient, additional inflows of resources from development partners in the form of budget support (Additional file 1).

As Ethiopia designs and implements its home-grown socio-economic policy response to COVID-19, it may wish to consider some key implications of the crisis that could shape the policy space available to it in the short- to medium-term**:**

It will take longer to delivery as response and recovery substantially complicate and delay previously planned trajectories and targets across the board. Opportunities will also emerge, however, to accelerate a return to the trend, for instance, using repurposing to boost MSMEs in the manufacturing sector through the production of health equipment, sanitizers, masks and supplies or to take a significant step forward in the digitalization of services in the public and private sectors.

## Opportunities of COVID-19 for some sectors

Beyond its innumerable challenges and crisis, Corona viruses has some advantages and opportunities for some sectors in the country. From these many firms are trying digital activities for the first time, opportunities will also emerge, however, to accelerate a return to trend, for instance, using repurposing to boost MSMEs in the manufacturing sector through the production of health equipment and supplies or to take a significant step forward in the digitalization of services in the public and private sectors and greatly initiating innovations.

## Conclusions

This paper demonstrates small business and their challenges during the corona virus pandemic in developing countries, specifically in Ethiopia. Using different empirical reviews, WHO and other organizations reports/assessments, magazines, small business experiences and other related secondary data analyses, the researcher tried to interpret analyze and make reasonable conclusions about each small business challenges in the current crisis time. This study finds that doing business in this COVID-19 pandemic time is very challenging and has a dangerous impact on small businesses, worker life as well as the country’s overall economy. This study has some limitations including that it used only secondary data, because it is difficult to get empirical/primary evidence directly from firms in this pandemic time; so it was better to include primary data. Furthermore, because of COVID-19 is a recent pandemic it was difficult to get more related literatures for review.

COVID-19 disruptions do not affect all businesses equally. Some are deemed essential and remained open, while others were required to close. Some businesses could shift employees to remote work, while others were ill equipped for the transition. In this section, my study results suggest that disparities will be larger if the pandemic ends up lasting for several months.

According to Fairlie (2020b) findings, African-American business owners were hit the hardest by COVID-19. The first estimates from April 2020 for black business owners in the United States indicate a massive drop of 41% in business activity. Black business owners were also disproportionately negatively affected in May and June relative to national levels with declines in business activity of 26% and 19%, respectively. Simulations indicate that the industry distribution of blacks was partly responsible, placing black business owners at greater risk of losses in business activity due to the pandemic. Latin businesses were also hit hard by COVID-19 losing 32% of active business owners in April, 19% in May and 10% in June. Asian business owners experienced a 26% decline in business activity over the critical 2-month window, and continued losses of activity of 21% in May and 10% in June. Simulation estimates also point to unfavorable industry distributions for Latin, but the evidence is less clear for Asians. Immigrant business owners were also devastated with losses of 36% of business activity in April. Continued disproportionate losses were felt in May (25%) and June (18%).

Although industry distributions placed some groups at higher risk of closures in the pandemic, differences in the scale of businesses are likely a major cause of disproportionate losses among minority-owned businesses, which are smaller on average (Fairlie and Robb 2008; U.S. Census Bureau 2012).

Larger businesses are more likely to have the resources, business and legal structure, and returns to scale to implement procedures to address social distancing regulations for operating and re-opening during the pandemic. However, in Ethiopia, there are more of small businesses operating and it was difficult to survive in this crisis time, even the government is not facilitating different supports and mechanisms to overcome this challenge like that of the above countries research finding showed.

While this research generates diversified important insights, future studies can conduct extensive surveys in line with the findings of the article to have a comprehensive understanding on the different problems/challenges of small business owners in patriarchal developing nations with the widespread coronavirus and other pandemics. Besides, as the secondary data analysis the study was conducted within 5 months of the identification of corona case in Ethiopia, future researchers can concentrate on gendered experiences at the later phase of the pandemic to investigate certain changes of small businesses experiences.

### Implications of the study

#### Theoretical implications

This study contributes to the existing literature in crisis management and identifies the survival and resilience strategies of small businesses during a long-lasting crisis and challenge. Basically my findings are aligned with those of other scholars works relating to this topic and is important to show/provide interesting and recent perhaps promising areas to work on under the crisis time.

#### Practical implications

The actual result of the paper also provide recommendations for small businesses on how to remain flexible or competitive through resilience and renewal strategies, and the researcher has also gave relevant recommendations for policymakers and other concerned bodies. As previous research has shown, the tourism and agriculture industry is highly vulnerable to public health crises (Irvine & Anderson, 2004, 2006; Jonas et al., 2011; Wilks et al., 2006). Thus, with an economy heavily focuses on tourism and peripheral industries, Macao needs to increase diversity. Small firms need to develop their capacity for pivoting and adapting their business models.

#### Policy implications

The major type of policy refers to the regulatory and licensing requirements of small service firms. Although small service firms operate with fewer policy restrictions, firms in these sectors are more prepared to deal with a crisis. Thus, firms’ preparedness suggests that expanding regulatory requirements to more service sectors may also help deal with a crisis. Finally, there is a need for policies and mechanisms that put the small firms in contact with a broader number and range of stakeholders, including specialists.

#### Suggestion for future studies

For the future it needs further research on the impact of COVID-19 on business performance, comparative studies on before and after crisis and also how to manage this kinds of pandemics and their challenge during crisis time.

#### Alignments of other findings with this study

Bartik et al. (2020) conducted a survey in late March of nearly 6000 small businesses that were members of the alienable business network. They find that 43% of businesses are temporarily closed, large reductions in employees, and the majority of businesses have less than 1 month of cash on hand.

These results build on the findings from a few related studies of the early effects of the coronavirus on small businesses in the world. Employer business applications as measured by the U.S. Census weekly Business Formation Statistics (BFS) fell in 5 weeks from mid-March to mid-April by over 27% relative to the previous year (Wilmoth, 2020). Examining more recent data from the BFS there is some evidence of a bounce back, but weekly estimates show a lot of variation (U.S. Census Bureau, 2020). Estimates from the weekly U.S. Census Small Business Pulse Survey indicate that roughly 50% of businesses report having a large negative effect from the COVID-19 pandemic and that only 15–20% of businesses have enough cash on hand to cover 3 months of operations (Bohn et al., 2020; U.S. Census Bureau, 2020). Another weekly survey indicates that decreased demand is more problematic than supply factors, such as accessing materials and goods (Desai & Looze, 2020). The Stanford Latino Entrepreneurship Initiative (2020) surveyed 224 high-revenue Latin-owned businesses and found that 86% of respondents reported immediate negative effects, such as delayed projects and closure from the pandemic. This paper builds on the previous work by focusing on early-stage effects in April–June using CPS data, and by exploring differential effects for female, minority and immigrant business owners, which is potentially important for targeting government aid to preserve small businesses and the jobs they create.

This research finding supported the above study results about the challenges and effects of COVID-19 on small businesses.

## Recommendations

This research’s findings recommends that the government of the country should make risk analysis and business steadiness planning in all critical federal and regional institutions, explore opportunities for cross-border digital trade, and cooperate with domestic small business firms, support medium- and long-term business investments that lead to resilient supply chains for critical goods and services under the market, accelerate development of e-commerce/e-marketing systems including digital marketing, digital banking, digital payments mechanisms, e-financial services (e.g., microcredit and micro insurance), a national ID system, and robust digital communications systems within government and other office activities overall the country.

COVID-19 outbreak widely impacts SMEs in the country and most studies showed that there is a lack of flexible work strategies, formal documentation mechanisms, and comprehensive crisis management and aftershock strategies for new micro enterprises with little previous crisis experience. We recommend that small businesses and owners of small business should consider long-term and adoptive crisis management strategies, not only focusing on financial factors but also fully taking nonfinancial factors into account, owners top management, as well as different levels of personnel, should be involved in developing crisis management mechanisms according to their needs. All should work in exploring new markets and incorporating technology into their future growth, produce different sanitary materials up to end of the pandemic, and make their marketing flexible based on situations, which is another sign of active learning and improvement from the crisis. In the long run, the urge to grow and expand market share may be a strong driving force for small business to strengthen their resilience and renewal strategies.

## Supplementary Information

**Additional file 1.** Givernment income support level for employees and small business firms durring COVID-19.

## Data Availability

Not applicable.

## Abbreviations

WHO: World Health Organization
MSEs: Micro and small enterprises
UNICEF: United Nations Children’s Fund
UNECA: United Nations Economic Commission for Africa
