# Supplementary figures and images for: Small businesses and their challenges during COVID-19 pandemic in developing countries: in the case of Ethiopia
Source: J Innov Entrep. 2022 Jan 10;11(1):1. doi: 10.1186/s13731-021-00191-3 (PMC8744037; doi:10.1186/s13731-021-00191-3)

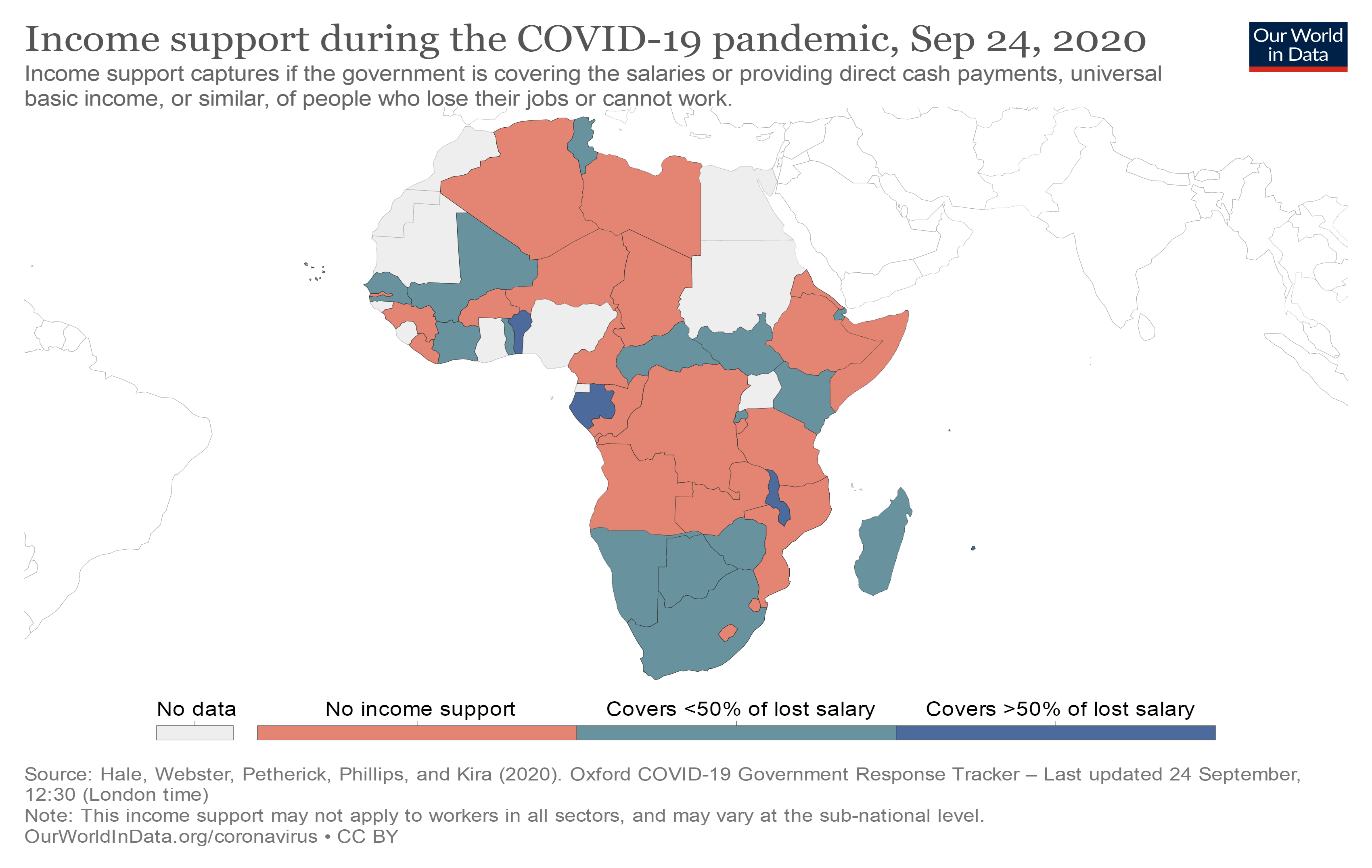

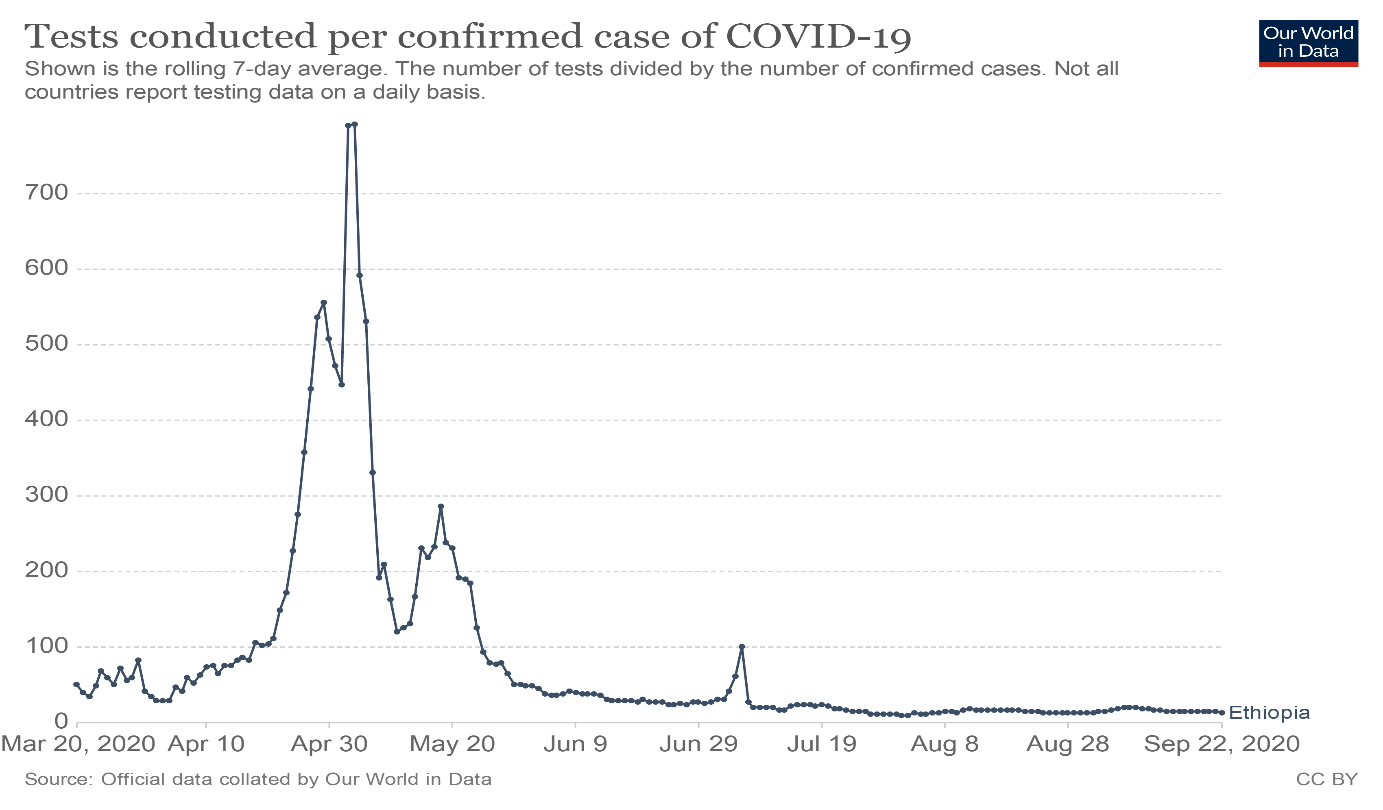

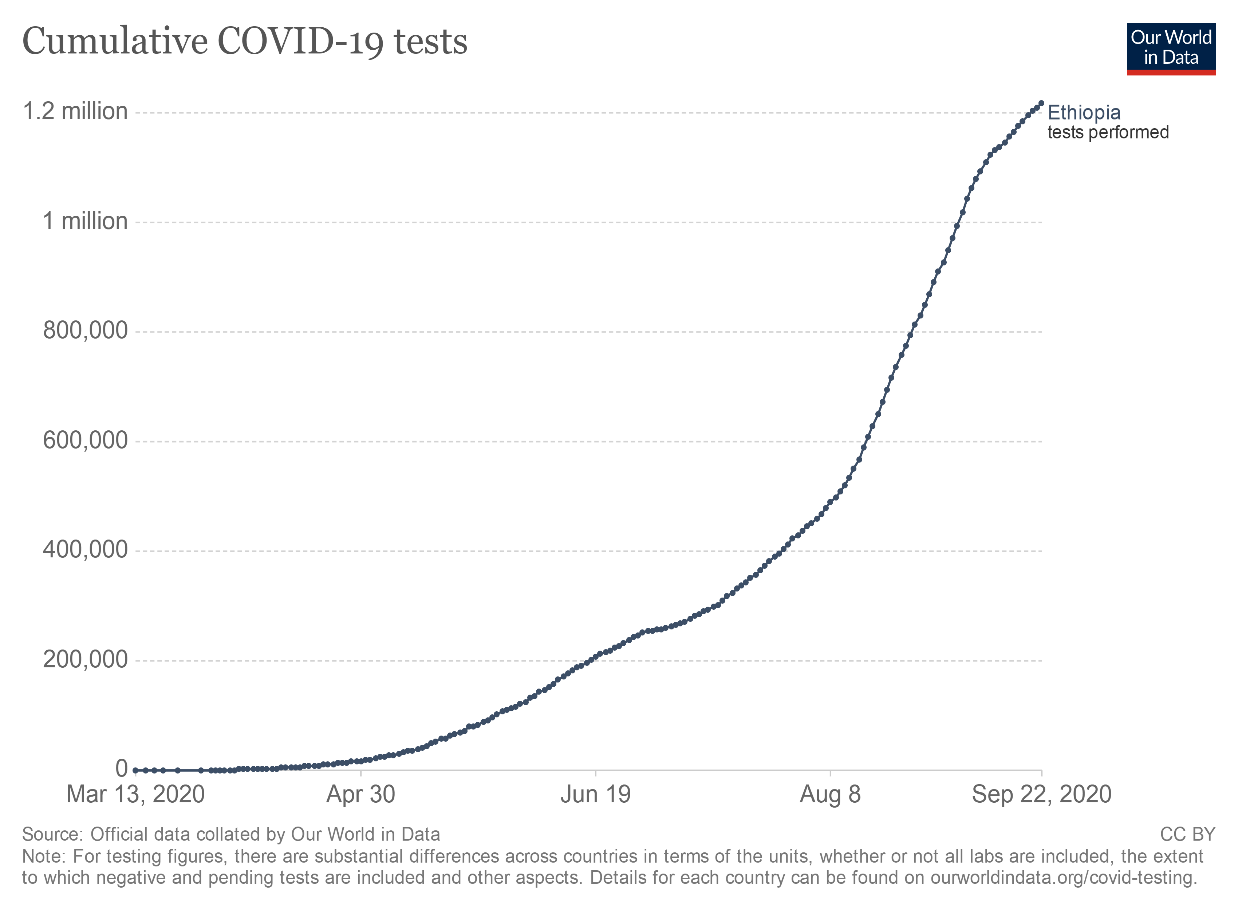

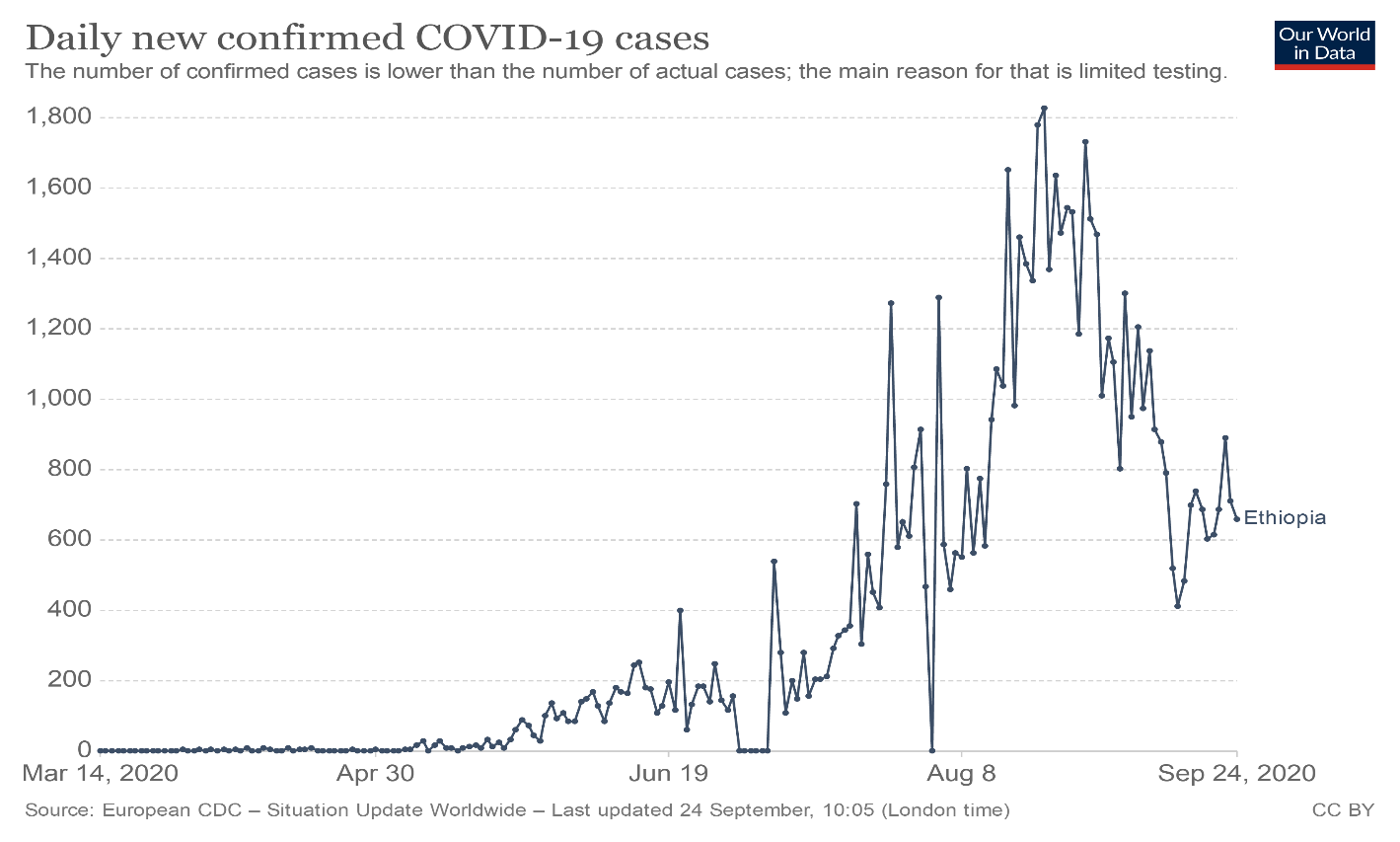

Supplement: Supplementary file 1 — Additional file 1. Givernment income support level for employees and small business firms durring COVID-19. [file 13731_2021_191_MOESM1_ESM.docx]
